# Co-creation, co-design or co-production? Reflections on the development of urban health systems implementation strategies to improve access and quality of primary healthcare services in Bangladesh, Ghana, Nepal and Nigeria

**DOI:** 10.1186/s12961-026-01467-4

**Published:** 2026-04-27

**Authors:** Helen Elsey, Francis Potier, Mahua Das, Sushil Chandra Baral, Rumana Huque, Deepak Joshi, Deepa Barua, Genevieve Aryeetey Okailey, Adanna Nwameme, Chinyere Mbachu, Lauren Jean Wallace, Selase Odopey, Delali Kumapley, Irene Agyepong, Obinna Onwujekwe

**Affiliations:** 1 University of York, Hull York Medical School, Seebohm Rowntree Building, Heslington, YO10 5DD UK; 2 University of Leeds, Leeds, UK; 3 HERD International, Kathmandu, Nepal; 4 ARK Foundation, Dhaka, Bangladesh; 5 University of Ghana, Accra, Ghana; 6 University of Nigeria, Nsukka, Nigeria; 7 Ghana College of Physicians and Surgeons, Accra, Ghana

**Keywords:** Co-design, Co-creation, Co-production, Health systems, Primary care, Urban health

## Abstract

**Introduction:**

Increasing populations and healthcare demand are leading to a burgeoning of private, nongovernmental and informal health providers addressing gaps left by overstretched public primary care and underresourced local government in urban areas in low and middle-income countries (LMICs). While evidence-based interventions exist to address common conditions in primary care, how to implement these interventions within complex urban health system is less clear. Enabling all relevant actors to feed their views and experiences into the process is seen as a key in co-design literature; however, the complexity of urban contexts makes planning and instigating such processes challenging. To inform future efforts to co-design system-wide approaches to implement existing evidence-based interventions in complex urban environments, we present reflective case studies from four cities in Bangladesh (Dhaka), Ghana (Accra), Nepal (Pokhara) and Nigeria (Enugu).

**Methods:**

We used the definitions and domains of co-creation, co-design and co-production from Vargas et al. 2022 to analyse reports of design meetings from each city and conducted four workshops where research teams involved in the design processes developed timelines of design activities and decisions and reflected on their interactions with stakeholders including: city authorities, communities, informal providers, ministry officials and public and private primary care providers. We coded reports and workshop outputs according to domains identified by Vargas et al.: focus, stakeholders involved, their role and level of participation, communication, value creation, resultant initiative and potential outcomes.

**Results:**

Key characteristics of co-production, co-design and co-creation were observed, often simultaneously, within each of the health system intervention development process. These categorizations varied by stakeholder (for example, city officials or communities) and at different points in the design process (for example, analysis or material development). The inclusion of locally generated research results was key in shaping and focussing the interventions and implementation strategies to ensure they addressed the realities of local health systems. Intense engagement with local government and health provider stakeholders facilitated their willingness to challenges and find appropriate solutions.

**Conclusions:**

Careful consideration of context, hierarchies among professionals, relationships between providers, underlying values and targeted use of locally generated qualitative and quantitative information to highlight gaps and strengths is key to developing implementation strategies to strengthen urban health systems.

**Supplementary Information:**

The online version contains supplementary material available at 10.1186/s12961-026-01467-4.

## Introduction

Many urban areas in low and middle-income countries (LMICs) have experienced rapid and unplanned growth. Despite global goals to achieve universal health coverage, the prospects of ensuring affordable and quality primary healthcare services in many LMIC cities are challenged by the limited availability, accessibility and scope of public primary care and community outreach in cities. Over half of all workers in urban areas in LMICs work in the informal sector [1], which means they frequently work long and unpredictable hours, making access to public primary care challenging [2].

The infrastructure of urban areas is under strain, particularly public primary healthcare services, which were not designed to meet the changing health needs of a large and growing urban population [3, 4]. There is also a rapid growth of urban slums or informal settlements, with a paucity of public healthcare facilities and other essential health services [5–7]. In addition, government primary healthcare services themselves face considerable challenges to extend and improve their services to address the changing disease burden with the rising prevalence of noncommunicable diseases as well as continued communicable diseases.

The low level of availability of public health facilities in low-income urban neighbourhoods has led to a burgeoning of both formal private, nongovernmental (NGO) and informal health providers in many urban areas [4, 8]. Working with these providers to improve the quality of care and provide linkages between types and levels of care is increasingly recognized as the only viable option for delivering universal health coverage in urban areas in LMICs [4, 9–12]. The range of providers can be extensive, and the boundaries between formal and informal are frequently blurred [9, 10]. For example, in Nigeria, research in Enugu city highlighted at least four categories of informal providers: (1) traditional birth attendants, (2) patent medicine vendors, (3) traditional and herbal medicine providers and (4) bone setters; each have their own system of organization, practices and belief systems and often have informal connections to the formal health system [11].

Establishing systems to link this plurality of providers to government health systems presents opportunities in reaching the urban poor [10, 13], but challenges are also well documented and include identifying providers, navigating registration and regulation processes and practices, working within existing policies and overcoming suspicion between public and private [11, 14]. Finding systems solutions that are appropriate to these complex urban environments, acceptable to such a wider range of stakeholders, which have the potential to be effective given the threats to health in urban areas, is a significant challenge. The best approach for finding contextually appropriate solutions that can be sustainably delivered in urban areas will be through the co-creation of such interventions and implementation strategies with stakeholders at the local level.

There has been significant work and insights on the process of collaboratively designing interventions and implementation strategies to address a wide range of healthcare and system challenges [15–19]. While several reviews identify a wide range of overlapping definitions of co-design, co-creation and co-production [17, 18], Masterson et al. (2022) recommend agreeing and being explicit about values and principles underpinning the process rather than focusing on defining concepts [17]. Vargas et al. (2025) highlight the following underpinning values and processes of co-design: collaborative and inclusive, actively involving diverse stakeholders; addressing a specific problem; creating innovative and relevant solutions through varying degrees of participation at different stages; and finding solutions that meet the needs and expectations of all participants [18] While implicit in Vargas et al.’s (2025) articulation, Masterson et al.’s (2022) scoping review explicitly highlights the active involvement of service-users as a key principle of co-design. Drawing on these key principles research teams in Bangladesh, Ghana, Nigeria and Nepal within the “Community-led Responsive and Effective Urban Health Systems” (CHORUS) research consortium embarked on a co-design process. The teams aimed to collaboratively develop health systems solutions by bringing together public, private, informal and NGO providers with government primary care as well as community members to improve health services for urban poor communities in Dhaka (Bangladesh), Accra (Ghana), Enugu (Nigeria) and Pokhara (Nepal). The health systems solutions focused primarily on primary care with a particular focus on bring access and services close to low-income urban residents. Given the gaps in public primary care provision, working with the private formal and informal health providers frequented by vulnerable and low-income populations was a priority for the co-design teams.

Successfully implementing a co-design approach within the complexity of urban health systems characterized by a plurality of public and private providers remains an underreported area. Vargas et al. (2022) make the distinction between co-production and co-design, which they situate within the overarching concept of co-creation, with co-creation referring to the most participatory process. Co-creation is described as a: “collaborative approach of creative problem solving between diverse stakeholders at all project stages to ownership” (Vargas et al. p. 2 [19]). This approach draws heavily on concepts of participatory action and research [20]. Many authors argue that co-creation has the potential to come up with new solutions that build on mutual learning across different stakeholders and are, therefore, able to mobilize collective energy and knowledge that is more likely to result in sustainable solutions [21–23]. Longworth et al.’s (2024) systematic review of co-design of public health approaches in LMICs found that most of the research focused on co-design with community members and patients, with only one study documenting co-production with city authorities [15]. In this paper, we present our experiences of co-creation to find solutions to the provision of accessible and acceptable quality primary healthcare services for low-income urban residents in West Africa and South Asia. We aim to provide reflections and lessons for researchers, policy makers and practitioners embarking on similar processes.

## Methods

Within the CHORUS programme, our aim was to co-create health system interventions that could be feasibly and sustainably delivered and that could also be assessed and studied within a viable research programme with clear research questions. In particular, our programme focused on four key urban health systems challenges: (1) working across the plurality of providers, (2) multisectoral action to address the wider determinants of health, (3) capacity to address both noncommunicable and communicable disease (4) and engaging urban poor. Attempting to balance the value of our work to the wider global evidence base, with the vital implementation outcomes within the city and health system context, was a considerable tension throughout the process. The recent growth in quality implementation research studies [24] proved valuable in shaping the research questions and design of research components to ensure that subsequent research could answer implementation questions of relevance to communities, city governments and local health providers as well as contribute to the wider evidence-base.

We drew on guidance from O’Cathain (2019) to help plan our co-creation phase, recognizing the importance of dynamic and iterative processes that include multiple key stakeholders but also the need to review existing evidence and theories, develop theories of change, collect primary data and understand local context [25]. Defining when the co-design process begins can in itself be challenging; however, Vargas et al.’s (2025) review presents a synthesises of the steps reported in published studies. This identifies a preparation phase which includes planning and participant buy-in and the identification of needs as the first two steps. In terms of planning and buy-in, in each city context, the CHORUS partners had long-standing working relationships with informal urban community leaders and city and health system decision-makers, and key individuals were involved in initial discussions to shape the focus of the health system challenges to be addressed. For all teams, the stakeholders identified as relevant to the co-design process were those that could represent or present the views of those who would either potentially be the target beneficiaries for the intervention or be involved in its delivery or management. In this initial preparatory phase, the research team’s intention was to gain buy-in of all relevant stakeholders to the co-design process, ensuring that they felt their further engagement in the co-design process would be of value to their own agenda as well as the wider agenda of strengthening urban health systems.

This early preparation phase also involved extensive needs assessments which included analysis of existing datasets, systematic reviews of the evidence base on public private partnerships in urban areas [26] as well as detailed qualitative, participatory and quantitative assessments of health providers and the health-seeking behaviours of urban poor households in the study cities (see Table 1). Given rapid and dynamic urbanization in all the case study countries, population and health system data are often limited or out of date. The intention of the needs assessment phase was therefore to address these gaps in essential local information to inform the co-design of the health systems interventions. The needs assessment frequently highlighted additional stakeholders, particularly community leaders and representatives of different provider groups (for example, associations of informal health providers) or NGOs to be engaged in the core co-design process, and the research teams would discuss the plans and aim of strengthening urban health systems with these new stakeholders to seek their buy-in.

**Table 1:** Summary of needs assessment activities that informed co-creation

	Ghana	Bangladesh	Nepal	Nigeria
Secondary data analysis	Analysis of health information system data from 29 districts to assess differences in MNCH services in slum and non-slum areas [27]	Analysis of the Bangladesh Health Facility Survey (2017) to assess diabetes and hypertension management in urban PHC	Analysis of urban population data (*n* = 3460) from STEP survey (2019) to compare social determinants, poverty with NCD risk and prevalence	N/A
Policy and literature review	Systematic review of Community Health Planning and Services (CHPS) implementation [28] Rapid policy review of CHPS	Rapid review of urban health and NCD policies and NCD and primary care studies conducted in Bangladesh	Rapid review of urban health and NCD policies and NCD and primary care studies conducted in Nepal	Scoping review of informal providers [10] and rapid review of Enugu state policies
	Systematic review of public private partnerships in urban LMIC contexts (in press)			
Qualitative and participatory methods	Extensive participatory approach: rich pictures with local government, health providers, and communities; transect walks in four urban poor communities Qualitative interviews: patients [24], health providers [14]; focus group discussions with community residents [14]	21 qualitative interviews with healthcare providers and policy makers 22 interviews with community members (men, women, transgender)	Two social mapping/transect walks with community gatekeepers Seven interviews with city officials and health providers Six focus groups with community members 10 interviews with patients with NCD	32 interviews with informal health providers 12 decision-makers, 16 PHC managers, 16 informal community leaders [11]
Quantitative assessments	Cross-sectional household survey (*n* = 3453); facility survey of 110 community health workers and observation of five CHPS compounds [29]	Cross-sectional assessment of NCD preparedness in 19 government urban dispensaries and 32 NGO clinics [30]	Cross-sectional assessment of 398 primary care and pharmacy NCD services [31]	Cross-sectional assessment of 254 formal and informal health facilities in eight slums across two states and 1025 households to assess use of formal and informal providers [32]
Focus of urban health systems intervention	Expanding and making CHPS responsive to the urban context through focus on NCDs (including hypertension diabetes, mental health) and reproductive health, strengthening health provider capacity and reshaping community engagement and outreach	Strengthening systems and health provider capacity to deliver hypertension and diabetes care and prevention	Training pharmacy staff for screening, health information, recording and referral and strengthening primary care for diabetes and hypertension	Establishing a system of linking informal providers to the formal sector through training, reporting and referral

The co-design process took between 12 and 18 months and involved workshops, formal and informal meetings and evidence-sharing. Details of all activities conducted during the preparatory and co-design phases can be found in the Supplementary Materials. CHORUS teams facilitated community, provider and other city actors to develop appropriate and feasible health systems solutions linking private, NGO and informal providers with public facilities and reshaping primary care to better meet the needs of deprived urban communities. Resultant interventions and implementation strategies included improved systems for referrals, supportive supervision, data recording and reporting as well as training and capacity strengthening of providers, implementation guidelines and materials for patients.

**Table:** 

**Box 1: definitions of co-creation, co-design, co-production adapted from Vargas et al. 2022 ([**19**] p. 2)**
Co-creation: collaborative approach of creative problem solving between diverse stakeholders at all stages of an initiative, from the problem identification and solution generation through to implementation and evaluation.
Co-design: active collaboration between stakeholders in the design of solutions to a prespecified problem.
Co-production: implementing previously determined solutions to a previously agreed problem with emphasis on the most efficient use of existing resources and assets.

We collated reports of the design meetings, developed timelines of the design process and held four reflective group discussions (one with each country team). We used Vargas et al.’s (2022) definitions of co-creation, co-design and co-production (see Box 1) to structure the reflective group discussions and to analyse the reports and reflections to understand the nature of the intervention development processes in each country. To structure our timelines, we drew on the six-step co-creation process proposed by Vargas et al. (2022) and further developed in Vargas et al. (2025), beginning with a preparation phase to (1) identify and gain buy-in of stakeholders and planning, (2) identify needs, analysing relationships between stakeholders, values and options for interventions, followed by a design phase to (3) define priorities, next steps and actions and (4) design the initiatives including goals, resources and assets. The final two steps involve (5) implementation and (6) evaluation [19]. While presented in a linear fashion, Vergas et al. recognize the iterative nature of these six steps.

**Fig. 1:**
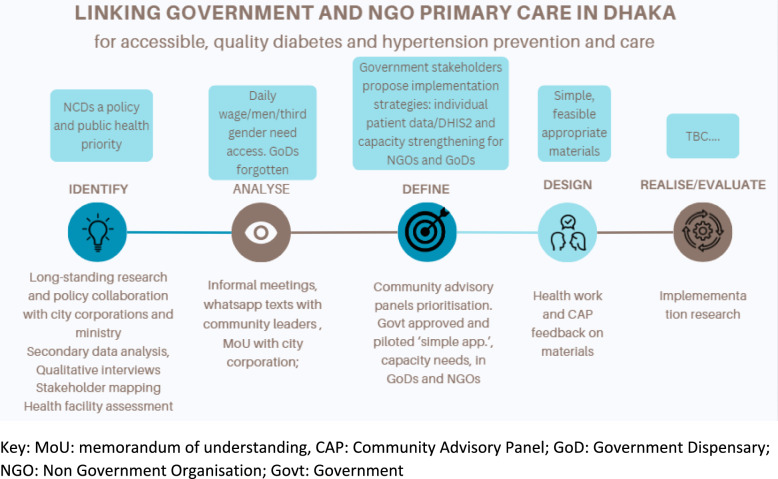
The design process in Dhaka, Bangladesh

**Fig. 2:**
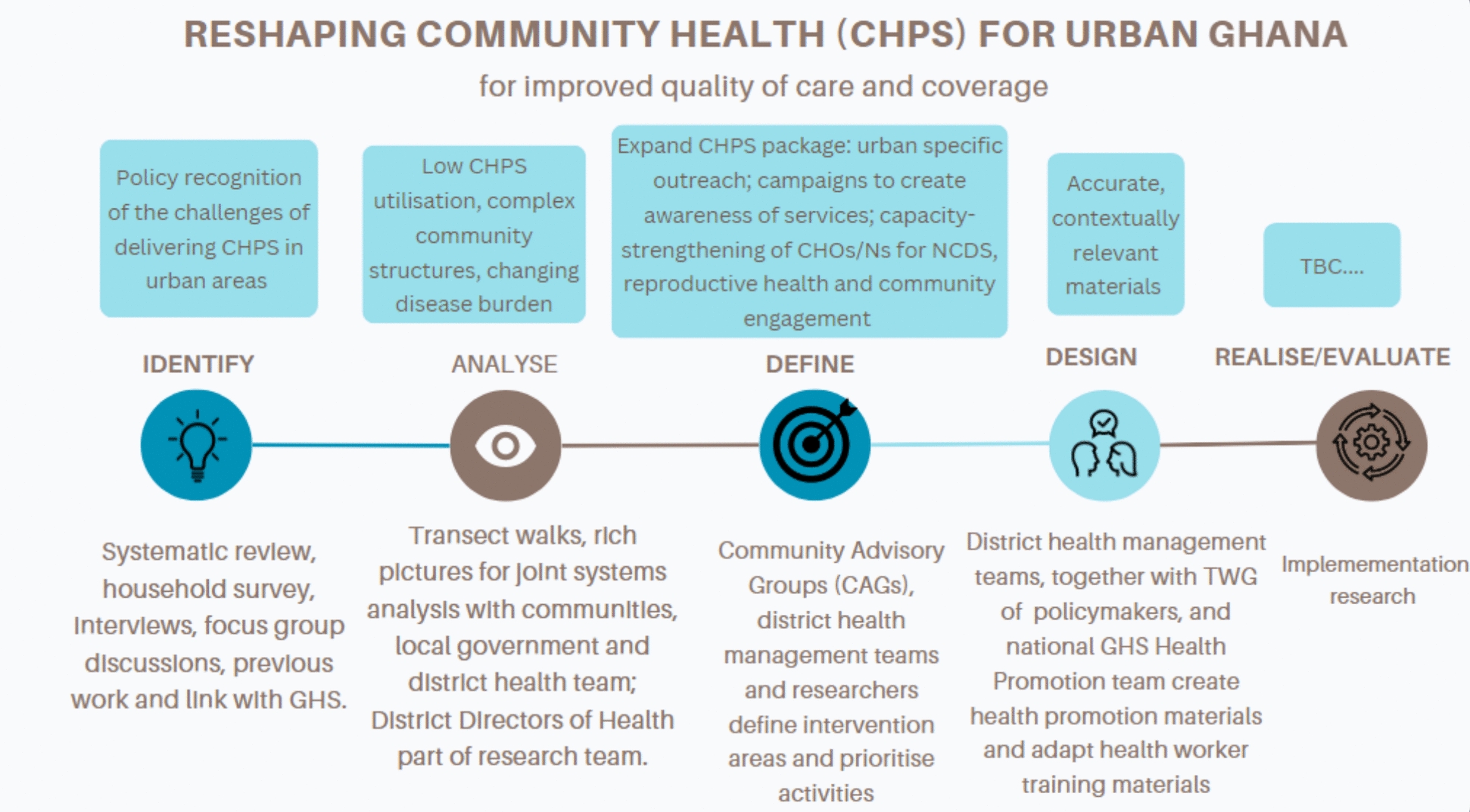
Design process in Accra, Ghana

**Fig. 3:**
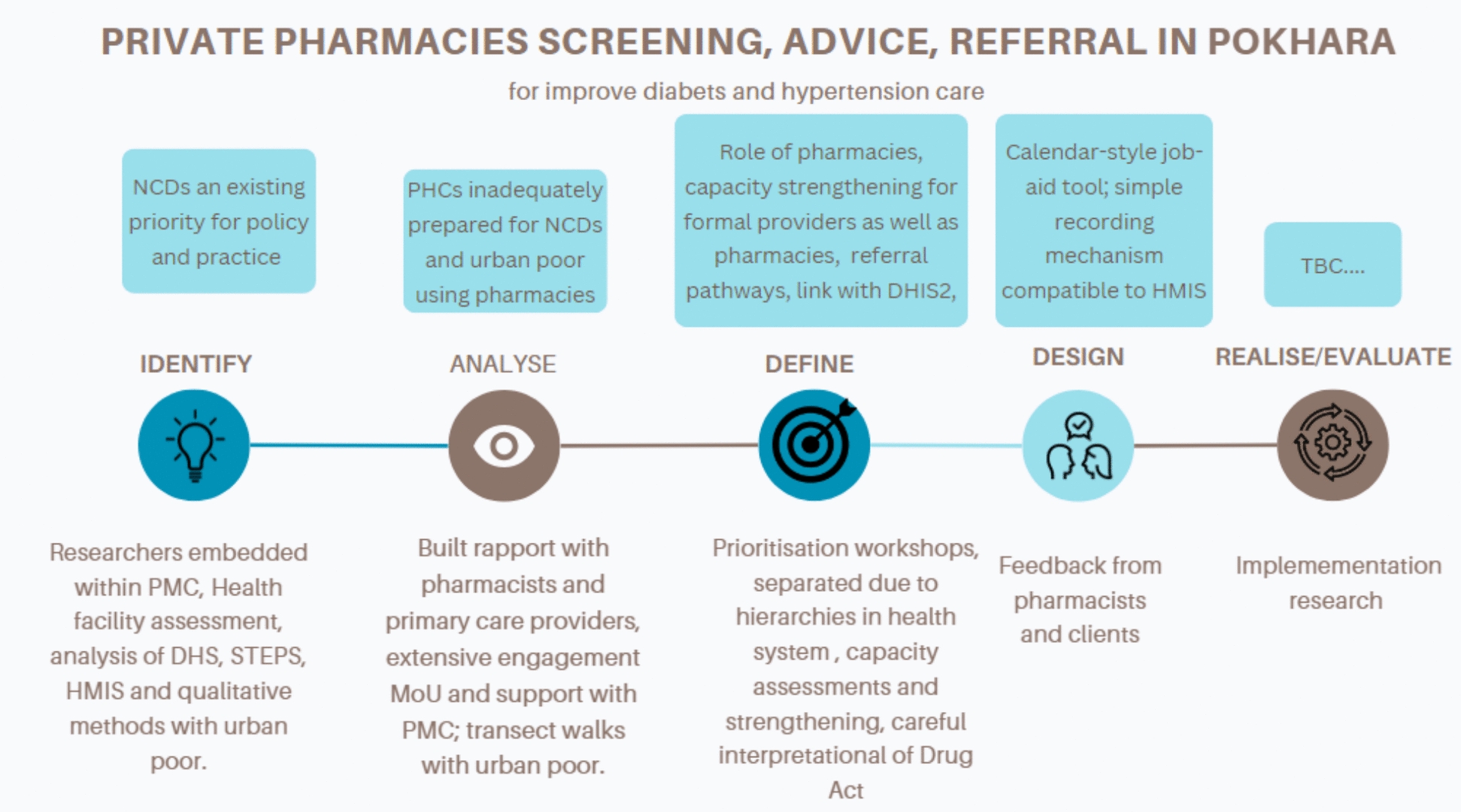
Design process in Pokhara, Nepal

**Fig. 4:**
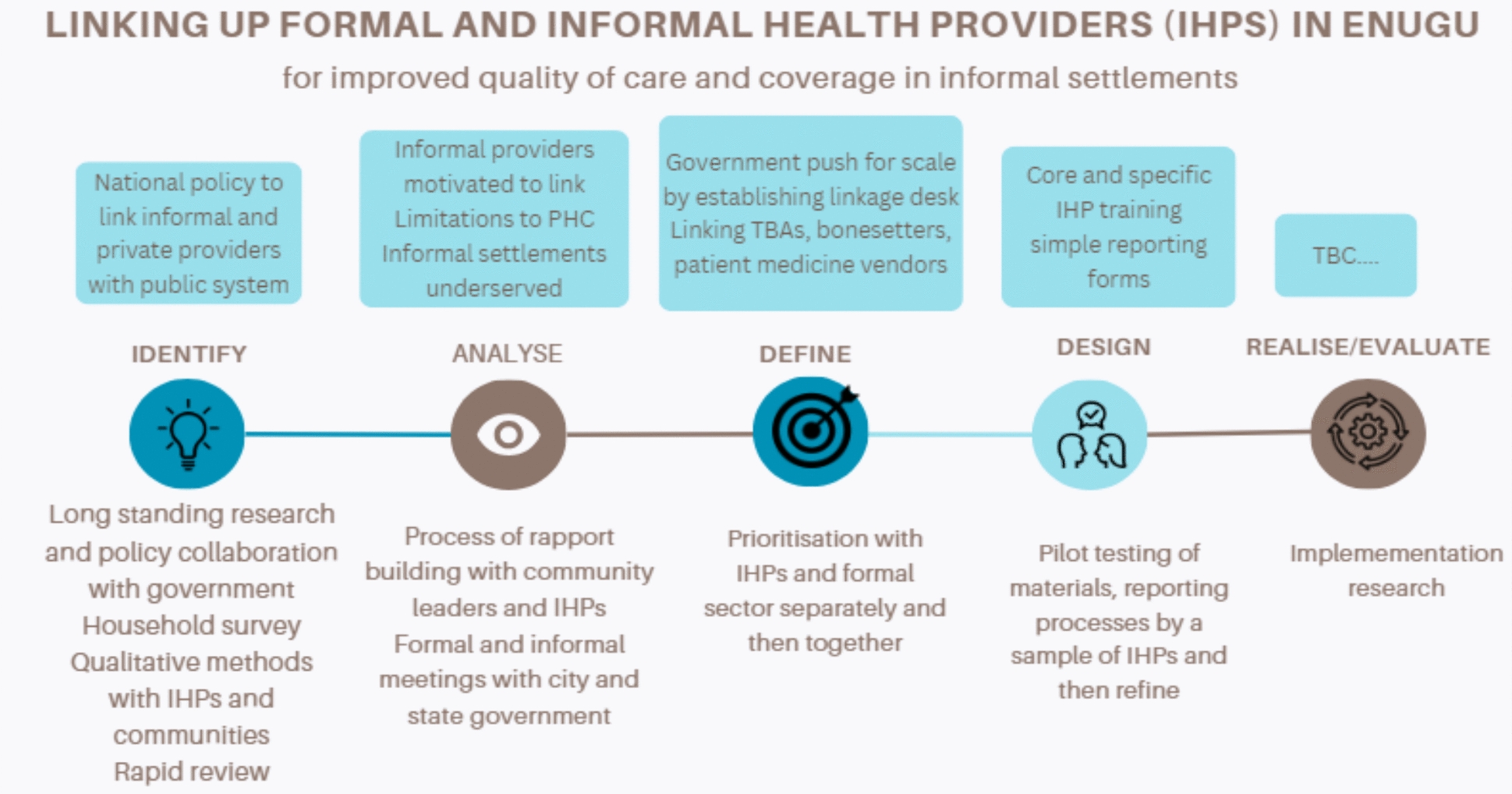
Design process in Enugu, Nigeria

Each case study below is presented according to the first four steps in this process, with the activities detailed below the line (in brown) and the decisions above the line (in blue) (see Figs. 1, 2, 3 and 4). Vargas et al. (2022) identify several aspects which help distinguish whether the process is classified as co-creation, co-design or co-production, namely: the focus, stakeholders involved, their role and level of participation, communication, value creation, resultant initiative and potential outcomes. We explored these dimensions within our analysis of the design process and team reflections. These dimensions form the basis for the themes presented in the results section. At the time of writing, guidelines for reporting co-design processes are under development [33, 34], and while we cannot pre-empt the content of the guidelines, we present here key aspects of the process, underpinning principles and details of the stakeholders and their engagement in an attempt to address concerns of the current lack of clarity in reporting of co-design processes [17–19, 23, 35].

## Results

### Case studies of the design process

#### Bangladesh: developing implementation strategies to improve noncommunicable disease (NCD) primary care

Introduction: primary care is struggling to meet the needs of an urban population growing at over 3% per year [36]. While a robust primary healthcare network exists in rural areas, primary care in urban areas falls under a complex governance structure of the Ministry of Local Government, Rural Development and Cooperatives (MoLGRD&C) and the Ministry of Health and Family Welfare (MoHFW). Given the large and increasing urban population and limited public facilities, urban primary care has been provided through a partnership of government and NGO clinics under the Urban Primary Health Care Project (UPHCP).

The UPHCP has been found to improve maternal and child health outcomes [37]; yet, addressing the rising prevalence of NCDs has added a new challenge for the primary care providers. While NCD corners delivering care according to the national protocol which is based on WHO’s Package of Essential Non-Communicable Disease Interventions for Primary Health Care (PEN) package [38] have been rolled out in government Upazila clinics in rural areas, such initiatives have been absent in urban areas. A detailed needs assessment (see Table 1) built an understanding of the current system and perspectives of health workers in public and NGO clinics, patients and communities. The needs assessment found limitations in the readiness of urban primary care (both NGOs and government dispensaries) to provide NCD screening, diagnosis, treatment and lifestyle support. The assessment also identified the lack of systems for front-line health professionals to record and report patient information on NCDs, particularly a consistent system across government and NGO providers. The needs assessment also found that several communities faced constraints in access; in particular, men were less likely to use primary care owing to the historical focus on maternal and child health, and the hijra, or third gender, rarely visited clinics owing to discrimination and stigma from health professionals and other community members. These findings were fed into the co-creation process.

Setting: the setting was the government and NGO primary care targeting urban deprived neighbourhoods in Dhaka.

Stakeholders: the stakeholder mapping conducted during the needs assessment, along with the long-term engagement of the researchers with the health system, allowed the team to identify relevant stakeholders to engage in the design process. These included relevant representatives from both city corporations operating under the MoLGRD&C; and representatives from Dhaka Civil Surgeon office, Upazila Health Care and Non-Communicable Disease Control (NCDC) Programme functioning under MoHFW as well as primary care health professionals from government dispensaries and NGO clinics. The intention here was to ensure not only that the co-designed intervention would fit within existing health systems but also that key decision-makers would champion the implementation of the intervention. Community leaders from informal settlements and low-income neighbourhoods were engaged through community advisory panels to give their perspectives on how to meet the diverse needs of community members. Development partners, including technical support from WHO, provided insights from previous work and guidance from similar contexts.

Engagements: formal and informal meetings were held with ministry, city corporation officials, development partners, NGOs and urban health academics as well as formal community advisory panels made up of community members, many of whom had lived experience of hypertension and/or diabetes. As shown in the timeline below (Fig. 1), through this process of engagement, the research team was able to share the findings from the needs assessment (see Table 1) and ask each stakeholder group to prioritize aspects to focus on in the intervention. During this process, government actors—particularly the Ministry of Health and Family Welfare—emphasized the need to focus on the government urban dispensaries. These dispensaries fall only under the MoHFW. The responsibility of NGO clinics operating under UPHCP fall under MoLGRD&C. However, these facilities must implement National Protocols issued by the MoHFW, therefore making both MoLGRD and MoHFW responsible for their operations. Both types of facilities are responsible for urban PHC. Their lack of inclusion in the Bangladesh Health Facility Survey sample highlights their invisibility within the system. The engagements also highlighted the importance of individual patient NCD records and data capture across different NGO clinics and public facilities such as the dispensaries. While NCD data from rural primary care were integrated and available with the national health dashboard, this was not the case for NCD data from any of the urban primary care providers. To address this, government stakeholders encouraged the inclusion of a mobile health application known as the Simple App. This has been piloted in rural areas and allows collection of individual patient data on hypertension and diabetes and is connected to the wider government health management informal system DHIS2. This led to the inclusion of the national heart foundation staff in the co-design process and the adaptation of the application for the urban context. The limitations in preparedness, particularly the lack of NCD-related counselling, follow-up and referrals, shaped the design of training to be delivered with government NCD experts, to improve NGO and public health providers’ knowledge and skills in NCD management in line with national hypertension and diabetes protocols. The community advisory panels gave feedback on all patient information on diabetes and hypertension developed to support improved delivery in line with national protocols and these were subsequently revised.

Intervention to be tested: it was strengthening systems and health provider capacity to manage and prevent hypertension and diabetes care and prevention by building the capacity of health providers and information systems in government and NGO primary care facilities to deliver screening, diagnosis, care and prevention to patients with, and at-risk of, hypertension and diabetes in line with national diabetes and hypertension protocols and WHO PEN package [38]. Providers use the Simple App to collect and use individual patient data. This is included in the national health dashboard to enables city corporations and MOHFW to analysis of NCDs in the city across government and NGO providers.

#### Ghana: adapting a community health programme for the urban context

Introduction: Ghana’s three-tier district health system has at its foundation the Community Health Planning and Services (CHPS) programme which aims to deliver universal access to health promotion, prevention and basic curative care in rural districts using community-based nurses known as community health officers and nurses (CHOs and CHNs) and volunteers [28, 39]. CHPS was successfully piloted in 1994 in rural Navrongo in the Upper East Region of Ghana [40] and rolled out on a national scale in 2000 [41]. The CHPS concept has recorded a lot of success in rural communities, including a reduction in childhood mortality [41] and declines in total fertility [42]. However, despite government policy to scale-up CHPS nationally, there are challenges in delivering CHPS services within complex urban environments [43, 44]. Ghana’s rapid urbanization is characterized by slum and peri-urban communities with poor infrastructure, overcrowded and unsanitary conditions with increased exposure to risk factors for both communicable and noncommunicable diseases leading to inequalities, poverty and marginalization [45] While urban communities may have greater physical access to a range of predominantly private clinics and pharmacies, access to quality, affordable services is limited, in part owing to limited uptake of health insurance, resulting in infant and child mortality five times higher in poor urban communities compared with the general urban population [45]. Within this context, CHPS, which relies on CHOs and CHNs living-in their communities and significant volunteer support, has struggled to reach urban deprived communities and provide the breadth of services that they now require, particularly to address noncommunicable diseases and mental ill-health [28, 43, 46].

Setting: the setting was four communities within two urban districts within the Greater Accra region: Ashaiman, which serves as a dormitory town from nearby industrial Tema and has many informal settlements and slum housing; and La Nkwantanang-Madina, also characterized by informal settlements and many migrants from the north of Ghana.

Stakeholders: at the community level, this included traditional and religious leaders, leaders of occupational groups/associations and residents. Given limitations in community engagement in many urban public services, the intention was to ensure that their current experiences of engaging with health providers and the CHPS programme were adequately captured in the co-design process. Community health workers, assembly members and district health teams were engaged to bring experience of delivering CHPS in the complex urban setting and to ensure the feasibility of co-designed interventions. National and regional policymakers from Ghana Health Service (GHS) and the Ministry of Health (MoH) were engaged throughout to ensure alignment with national policy and to generate champions of the new urban CHPS model at national level.

Engagements: given the need to adapt the rural CHPS model to respond to the realities of low-income urban residents, the team in Ghana ensured that community engagement underpinned the design process. Five stakeholder engagements were held (2020) to enable a series of rich pictures to be drawn, one for each of the four communities to understand the interacting facilitators and barriers to CHPS implementation in deprived neighbourhoods. District -level managers, including district directors of health, CHPS coordinators and local government staff for the two selected districts, were trained by the research team on rich picture development. Managers then engaged community members to develop rich pictures [47] from their own perspectives, building on the initial pictures, with guidance from the research team. These early engagements and subsequent joint analysis (researchers and district directors of health) of the rich pictures using systems thinking [47] informed quantitative and qualitative data collection in the needs assessment (2021–2022) (see Table 1). Findings of the needs assessment were then disseminated in each of the communities through separate fora; each forum was attended by approximately 50 people comprising community members, local government officials, health personnel including members of district health management teams, CHOs/Ns and CHVs, traditional and religious leaders, and the media. During these meetings, priority areas of intervention for the restructuring of CHPS in the urban setting were identified. These were then discussed through meetings with community advisory groups (CAG) (October–November 2023). One CAG was formed in each district composed of staff from district-level health teams, community health workers and community leaders. During meetings, the researchers facilitated the use of a participatory ranking approach where CAG members first individually ranked activities under each intervention areas before a finalized comprehensive group ranking was developed by the research team and agreed on by the CAG. This led to the agreement on the adaptations to CHPS to be trialled.

Following the initial discussions between CAGs and researchers, a technical working group (composed of national and regional health policymakers from Ghana’s GHS and MoH) was formed to assist with the development of the training package for CHOs/Ns, and health promotion materials to promote the expanded urban CHPS program to be used in the intervention sites, to ensure their technical and contextual relevance. Ghana Health Service’s Health Promotion Division also collaborated with researchers and TWG members to develop health promotion materials (February–November 2024). Feedback on the materials and messages was gained through the so-called dipstick approach used by GHS through focus groups with community members. Materials such as posters and mobile van broadcasts featured the pictures and voices of community residents from the intervention sites.

Intervention to be tested: expanding and making the evidence-based CHPS package [28, 41, 48] responsive to the urban context through a focus on strengthening the capacity of providers to address NCDs (including hypertension, diabetes and mental health) and reproductive health and reshaping community engagement and outreach.

#### Nepal: linking pharmacies to primary care to address hypertension and diabetes

Introduction: with federalization, metropolitan cities in Nepal now play a lead role in promoting, protecting and delivering health services to their urban populations. These new responsibilities came at a time of rapid urbanization and a change in the disease burden with growing prevalence of diabetes and hypertension. A recent prevalence survey found that those living in urban areas have almost two times the odds of having type 2 diabetes than their rural counterparts (adjusted odds ratio, AOR: 1.7, 95% confidence intervals, CI: 1.4–2.2) [49], and a meta-analysis of survey data found the prevalence of hypertension to be 28.4% (95% CI 22.4–34.7), 25.5% (95% CI 21.4–29.8) and 24.4% (95% CI 17.9–31.6) among urban, suburban and rural populations, respectively [50]. As the second largest city in Nepal, Pokhara has seen considerable population growth from 252,000 in 2010 to 494,000 in 2024 [51]. This coupled with the changing disease burden is putting great strain on the limited government primary care facilities. Given these challenges, the Pokhara Metropolitan City (PMC) health division were keen to work with the research organization, HERDi, to support their remit of improving urban health. In recognition of the need for technical support, researchers from HERDi were embedded in the health division of PMC not only to ensure their research was grounded in the realities facing the city authorities but also to provide necessary technical support to PMC’s health division including aligning implementation strategies with WHO’s evidence-based PEN package [38].

Setting: the setting was Pokhara Metropolitan City. The health facility needs assessment covered all primary care and pharmacies across the city. The design process focused on primary care and pharmacies in five wards of the city with six public primary healthcare facilities and approximately 30 pharmacies.

Stakeholders: PMC, health professionals and managers from the referral hospital and from primary care were engaged throughout to ensure that the co-designed intervention would fit with current health systems, particularly in the design of referral and supervision systems between pharmacies and public facilities and to nurture champions keen to promote the intervention Members of the Nepal Chemist and Drug Association (NCDA), and pharmacists were engaged to ensure that the intervention would be in accordance with legislation and regulation on the role of pharmacies and to ensure buy-in from local pharmacies. Ward chairs (elected) and community leaders and residents were engaged to ensure that the experiences of urban communities and their preferences for accessing services shaped the co-design of the intervention. Engagement: initially, officials at PMC were interested in knowing if the HERDi team would directly fund human resources or health services. However, during the so-called analyse phase (see Fig. 2), PMC became increasingly aware that information on the extent to which facilities were able to provide NCD services was limited, particularly to meet the needs of the urban poor. HERDi originally planned to only survey a sample of primary care and registered pharmacies in Pokhara; however, following a series of consultations, PMC requested the surveying of all pharmacies and PHC clinics. After some research budget refinement, a study plan for a census of these facilities was approved by the Municipal Project Advisory Committee, and a Memorandum of Understanding (MoU) between HERDi and PMC was signed in March 2022. This enabled the implementation of the NCD preparedness survey, mapping of urban poor settlements, qualitative interviews, and an analysis of PMC budgets (see Table 1). HERDi’s findings were presented at PMC Health Division’s annual review meeting and clearly highlighted that while not dispensing drugs, the majority of private pharmacies were offering clinical services for hypertension and diabetes and were the first contact point for low-income, daily-wage earners needing NCD services. In light of this, the research team was keen to develop a health systems intervention that could provide a link between pharmacies and primary care for optimum identification, advice and referral of those with (or suspected) diabetes and/or hypertension. Yet, the public sector felt that the provision of primary care is a core government role, and therefore, both PMC and primary care practitioners were initially hesitant to build linkages with the private pharmacies. Presentation of the evidence of the high utilization of pharmacies helped in part to overcome this; however, the HERDi researchers were very aware of the need to ensure a careful and extensive intervention design process. The team were also careful to use the word ‘linkage’ rather than any more specific term given the novel nature of the proposed system changes. A thorough review of relevant pharmaceutical acts clarified that the proposed new role for pharmacies to provide advice, screening and referral did fit within the legal framework, and this further helped to reassure PMC. The survey had also highlighted the limited preparedness of government primary care to deliver NCD care services. The systems analysis conducted as part of the design process highlighted the potential negative loop if pharmacy clients were to be referred to public facilities where they might not receive appropriate care for their diabetes and/or hypertension.

To shape the systems intervention within this context of limited capacity within primary care and unease at working with the private sector, the team held eight workshops with community members (divided by gender), community health volunteers, primary healthcare professionals, pharmacists, health facility operation management committee (HFOMC) and ward chairs. In addition, frequent meetings and phone calls were held with key individuals within PMC and the wider health system. Despite initial scepticism from the public sector, the intervention linking pharmacies to the primary care system was ultimately embraced by both pharmacists, PMC and primary care managers and practitioners. Pharmacists recommended which materials would be useful to support them to provide advice, for example, a calendar format and providing information for clients at the pharmacy and a leaflet to take home, and the content was revised by the officials from health division and PEN package [38] facilitators. Given the limited preparedness of public facilities to respond to NCDs, with technical assistance from CHORUS, PMC conducted PEN training for 144 health workers, including health assistants, staff nurses, auxiliary health workers, auxiliary nursing midwives, medical officers, Kaviraj and Vaidya, covering all 49 public health facilities (including a municipal hospital, health posts, urban health promotion centre, urban health centre and Ayurved Ausadhalaya). This training took place in three batches from January to March 2023.

Team reflections on the process highlighted the importance of the embedded research approach in building trust within PMC and with primary care health professionals. The use of evidence, analysis of the legal framework, flexibility in the needs assessment design further helped to keep the public sector engaged and ultimately keen to implement the intervention.

Intervention to be tested: the intervention was training pharmacy staff to provide diabetes and hypertension advice, screening and referral to strengthened primary care centres.

#### Nigeria: linking informal providers to primary care for improved health in informal settlements

Introduction: the Government of Nigeria recognizes the importance of a plurality of providers in delivering health care. This is articulated in the National Health Policy of 2016 which recommends the integration of providers through the Primary Health Care Under One Roof (PHCUOR) scheme. The policy is particularly relevant in urban settings where the paucity of formal healthcare providers, especially the public sector has triggered a rapid expansion in the market for informal healthcare providers (IHPs). The burgeoning of the informal healthcare sector is highly visible in and around poor urban neighbourhoods and informal settlements where nonformal and frequently unregistered healthcare providers such as patent medicine vendors (PMVs), traditional birth attendants (TBAs), bonesetters and traditional healers account for a significant proportion of health service delivery [52, 53]. The growth in IHPs reflects the high level of trust residents in informal settlements have in their services and advice as well as their convenient location and opening hours which, unlike the public sector providers, are suitable for those working long hours in the informal sector. Ensuring and improving the quality of treatments, advice and facilitating appropriate referral among these informal providers is key to improving the health of low-income urban residents as well as addressing key public health challenges such as inappropriate prescription of antibiotics and other medications.

Setting: the setting was Enugu state, in four informal settlements (slums) that are located within the metropolitan Enugu city. The informal settlements are spread across three local government areas. Two of the informal settlements are located within Enugu north LGA—Umunevo, Afia nine and Ngenevu, Obed camp; one is in Enugu east LGA—Ugbo Oghe, Abakpa; and one is located in Enugu south—Ikirike camp 1 and 2.

Stakeholders: informal providers including traditional birth attendants, bonesetters, patent medicine vendors, traditional healers were engaged. Their engagement was seen as crucial to ensure that the co-designed intervention was acceptable to them and built on their own motivations for engaging with the public sector. The rationale for including primary care health workers and managers, local government health authorities and regulatory agencies was to ensure that the public health system would be willing and able to accept referrals from and provider supervision and support to the informal providers and that the system could be feasibly delivered in practice. Community leaders, including members of women’s and youth groups, were sought as key participants, bringing insights on health-seeking behaviour in the informal settlements as well as shaping a mechanism for community members to hold both informal and formal primary care to account.

Engagements: the intervention design process built on a long history of interaction between senior academics and government officials on the role of the informal sector, in line with government intentions to integrate informal and formal health providers. This helped to shape the early reconnaissance work, which used qualitative methods and community engagement in eight informal settlements [11]. This participatory work tapped into the community structures in each informal settlement and explored in-depth the motivation for informal providers to engage with the public sector. The participatory nature of the interactions created an appetite among community leaders to explore new ways to improve healthcare within the settlements which facilitated engagement with key informal and formal providers. The first formal intervention design workshop (February 2023) brought together all stakeholders including the informal providers to share and reflect on the findings and recommendations from baseline assessment and to deliberate and agree on the potentially feasible interventions that could be implemented linking informal and formal providers. In this first workshop, 31 informal and formal providers were organized in separate groups to ensure different perspectives were heard. The groups generated ideas for possible interventions, which were then individually ranked and presented with a plenary discussion on feasibility and any adaptations of the top-ranked interventions. At this point, government actors involved in the process agreed to redeploy staff to establish a desk to support and oversee the linkage intervention. The research team then held a series of informal meetings and discussions with all groups of stakeholders to further develop the proposed intervention and to develop draft protocols and tools for the intervention. This culminated in a second workshop with all stakeholders, the majority of whom were also present in the first workshop, to review and validate a logical framework for each aspect of the intervention and to develop, adapt or review protocols and tools for implementation.

Given the importance, both in terms of policy and population health, given to linking informal providers to the formal healthcare system, government actors involved in the design process were eager to identify ways to institutionalize the linkage system developed. This led to the decision by the state health department to establish a permanent desk responsible for establishing and overseeing the linkage of informal providers. The research team worked with government actors to develop the specification for the two new posts created within the linkage desk. The research team will liaise closely with the new linkage officers during the implementation research which is planned to evaluate the linkage intervention.

Intervention to be tested: establishing a system of linking informal providers to the formal sector through training, reporting and referral based on existing national protocols of infectious diseases, maternal and child health and noncommunicable diseases.

### Reflections on the domains and categories of the design process

The categorization of the design process according to the Vargas et al. domains is presented in Table 2. The table illustrates how examples of co-production, co-design and co-creation can be seen simultaneously within each development process and that these categorizations varied by stakeholder (for example, city officials or communities) and at different points in the design process (for example, analysis or material development).

**Table 2:** Examples of co-creation, co-design and co-production from reports and reflective workshops

Vargas et al. classifications and characterizations	Bangladesh	Ghana	Nepal	Nigeria
Co-creation: collaboration of diverse stakeholders at all project stages, co-initiation calling for collective action: Characterized by: • All stakeholders involved and very active • Wide value creation • Engagement at all stages • Ownership of intervention by all stakeholders	Long-term formal and informal engagement with MoHFW and city corporations shaped the focus on NCDs and primary care and particularly the inclusion of both NGO and government dispensaries with use of an application already pilot tested and utilized by MoHFW Wide range of stakeholders involved including donors and National Heart Foundation who had led application developing and piloting. Community advisory panels established in informal settlements for long-term engagement	Participatory methods of transect walks and rich picture analysis designed with the input of district directors of health and then conducted with community members and health workers to shape which problems the intervention would address Bidirectional communication increasing over time with community and national technical advisory groups established for long-term engagement	Bidirectional transparent and ongoing communication with local government public health team due to research embedded within local government system Pharmacists and primary care health professionals involved throughout Careful and continuous engagement with pharmacies in their own settings helped to build trust and to understand their perspectives	Long-term formal and informal engagement with state health and primary care departments shaped the focus of the intervention to align with policy for integration of private and informal providers State government established an integration desk with two seconded officers to implement the intervention allowing long-term engagement with the research team
Co-design: active collaboration to address a prespecified problem, participation to improve a programme. Characterized by: • Social inclusion • Inclusion of lived experiences • Active role for stakeholders • Research team take a strong role in identifying the intervention but stakeholders co-lead design of modalities	Sharing of needs assessment findings with all stakeholders and facilitating them to identify solutions. To ensure lived experiences added value, the team sought opinions from people with diabetes and hypertension through their inclusion within co-design workshops, using separate focus group discussions to allow clear articulation of their perspectives	Participatory methods allowed the team to identify individuals within the community to improve social inclusion. This led to identification of multiple challenges Research team had to take key decisions on which intervention areas to focus on and engaged all relevant stakeholders in priority setting to identify and rank the top three options under each intervention area	Participatory methods and analysis of secondary data allowed researchers to identify use of private providers by urban communities, and therefore, researchers took a leading role in shaping the focus of the intervention Embedded researchers ensured a high level of trust allowing strong government and health provider buy-in despite nervousness of working with private sector Separate group discussions with front-line health workers allowed inclusion of their views despite health system hierarchies	Findings from the needs assessments were presented during workshops and each stakeholder group provided insights and recommendations for the design of the intervention. These were then shared and discussed with all workshop participants, and a modified Delphi approach was used to reach consensus on the most appropriate modalities and organizations/staff to deliver the intervention
Co-production: engages stakeholders in implementing a previously agreed solution. Characterized by: • Listening • Researcher driven • Value is enhanced beyond that pre-envisioned • Intervention is broadly prespecified • Stakeholder participation is likely to be passive and towards the end of the process	Gaining feedback from community members and patients on intervention materials Inclusion of transgender community only through research not active engagement	National and District GHS Health Promotion Teams developed awareness-creation materials, for example, poster and sample jingle. The GHS dipstick method using four group discussions with urban slum community members of diverse age, occupations and gender to obtain feedback on clarity, suitability and cultural relevance of materials. Materials were adapted on the basis of feedback. Final materials used pictures and voices of residents from intervention sites and were approved by GHS	Feedback from pharmacists on intervention materials and recording and reporting forms	Testing all recording and report forms with a small number of informal providers for their feedback and adaptation

#### The focus: balancing stakeholders’ views and evidence

The case studies highlight how the ultimate focus of each health system intervention was shaped by different stakeholder groups within each context. We identified three key factors which had a strong influence on the focus of the intervention: (1) the strength of relationship between the researchers and government actors at city and national level, (2) the use of evidence from secondary data analysis and existing literature and (3) the use of participatory methods with communities.

In Dhaka, the high degree of trust built through pre-existing long-term relationships with the MoHFW as well as with city corporations directed the focus of the intervention to include government dispensaries as well as NGO primary care clinics under the UPHCP. This underscores the importance of buy-in of stakeholders in the preparation phase, although in reality, the researchers in Dhaka have worked with government officials over many years on multiple studies in primary care. This deep engagement highlights the difficulty in initiating a co-design process with only a brief preparatory period to gain buy-in. The inputs of these government stakeholders fundamentally shaped all aspects of the intervention. For example, by identifying the value of not only training and logistics for improved diabetes and hypertension care but also implementing a data system, the Simple App became a core component of the health systems intervention. This data system was seen as the glue to link NGO and government clinics and provide the Ministry and city governments with routine data on NCDs in urban communities for the first time. Similarly, in Enugu, the long-standing relationship between the senior researchers on the team and the state and city authorities shaped the focus of the intervention in line with government policy to integrate informal providers within the primary care system. The detailed understanding of not only the policy environment but also the dynamics within the state departments shaped the focus to establish a desk within the state health department with agreement for the secondment of two salaried community health professionals to coordinate the process of integrating informal providers.

In Accra, the relationship between researchers, local governments and stakeholders from GHS was not as well established at the start of the project as it was in Dhaka. However, the limitations in this initial preparatory phase in creating strong buy-in were address by the research team through the use of a highly participatory process with communities, health workers and district health management teams and local government authorities. By using participatory methods such as transect walks and rich pictures, relevant stakeholders including health workers and community members could hear each other’s perspectives. This shaped the focus of the design phase to address both service-level issues as well as the main concerns facing urban residents.

In Pokhara, the focus of the intervention evolved as insights from engagements with urban poor communities—again using participatory techniques such as transect walks and ranking—were combined with views from pharmacists and healthcare providers and city government staff. The researchers also conducted an analysis of existing survey data to understand the use of private, pharmacy and public primary care for diabetes and hypertension. This analysis clearly showed high use of pharmacies and private clinics which aligned with messages coming from the communities. However, public health professionals in local government and primary care expressed concern at integrating pharmacies within care pathways for diabetes and hypertension owing to their profit motivation. The trusting relationship built up with the public sector was key in enabling the research team to address and overcome this tension through open discussion. Within these discussions, the presentation of survey evidence of the high use of private pharmacies was particularly powerful in gaining the agreement of the public sector to work with the private sector. The evidence also shed light on the limited capacity for NCD care within the public system and having relevant local data was key in reaching agreement not only to support private pharmacists to provide screening, advice and referrals for diabetes and hypertension but also to build capacity within the public sector to provide appropriate NCD services once pharmacy clients were referred.

#### Stakeholders’ roles and participation

Across the four case studies, the role of stakeholders and the extent of their participation differed at different points in the design processes. For example, in Ghana and Nepal, the initial participatory work with communities in poor urban neighbourhoods was key in highlighting the realities of accessing primary care. These perspectives are contrary to assumptions of a so-called urban advantage due to physical proximity to many hospitals and clinics [54, 55] and proved valuable not only in informing the focus of the interventions but also in identifying community members who may not have ordinarily joined intervention design workshops. In Accra, the views of working-age men, substance abusers and those with experiences of mental ill-health were heard through these participatory activities and could well have been missed without this on-the-ground engagement.

The challenges of engaging socially excluded stakeholders were particularly apparent in Dhaka. The researchers used qualitative methods with members of the transgender hijra community to understand their health-seeking behaviour. Their findings highlight the pervasive stigma that the hijra face not only from healthcare providers but also from the general population using primary care facilities; as a result, this population used only one pharmacy where the provider was prepared to treat their health needs. Since stigma of the hijra is so pervasive, the research team decided that inclusion of the transgender community within design workshops could be counterproductive and that a separate, targeted intervention co-created with this community would be needed, and further resources would be required for such a process. The experiences of this community were shared during the design process; however, there is a clear recognition that more needs to be done to overcome the high level of stigma which undermined their direct engagement in the co-design process.

All the case studies highlight how the role of stakeholders and their level of participation differed at different points in design timelines. The role of community members appears to cluster around the initial problem-identification stage and providing feedback on intervention key components, materials and key messages. For example, in Ghana, the focus of the intervention was driven by the insights of communities on their challenges in accessing primary care. However, it was the interaction with the district health directors, their teams and national stakeholders within Ghana Health Service (GHS) that proved more influential in coming to agreement on the parameters and shape of the implementation strategies to fit within existing systems. Focus groups of communities then provided feedback on already-drafted intervention materials and messages for the awareness-raising campaigns. The design process also highlights the evolving relationship between the researchers and the GHS, which grew considerably throughout the process, with GHS health promotion team working together with researchers to design and test materials using their own so-called dipstick methods.

In Enugu, the initial health needs assessment conducted in four informal settlements provided valuable insights and information in the services provided by informal providers and their motivations for integration (or not) within the public system. Importantly, in addition, the process of conducting the needs assessment allowed the team to understand the structures within the communities and to build rapport with community leaders and with informal providers. Qualitative methods were particularly important here as they allowed these stakeholders the rare opportunity to voice their experiences; this built rapport and trust with the research team. Within this qualitative work, the team were unable to secure interviews with several of the informal providers, and the team reflected that this was due to a distrust of the formal health system and authorities which illustrates the challenges of engaging with these stakeholders [56]. However, most informal providers and community stakeholders were willing to engage in the design process. The research teams’ understanding of these dynamics guided their facilitation of the design workshops. For example, workshops were organized so that informal providers and community stakeholders were able to discuss and form their recommendations before proceeding to plenary discussions with the public health professionals and city authority staff.

#### Communication

The reflections from all the research teams highlight the need for continual engagement with stakeholders using both informal and formal methods. For communities, this meant multiple visits, phone calls, and in the case of Bangladesh and Ghana, establishing and facilitating community advisory groups that would meet regularly. These groups often discussed issues such as contamination of the water and air pollution that were clearly wider determinants of health but tangential to the specific intervention under design. As the research team from Bangladesh explained, this was vital for building trust and reassuring communities that their inputs would lead to service redesign. This was a particular necessity in urban informal settlements who are often engaged as part of research projects but who, despite this, frequently see limited health improvements in their communities. To be able to hear all voices requires careful planning and participation approaches (Nepal).

A pre-existing and reflective understanding of the power dynamics between different stakeholders was a vital attribute of all the research teams. For example, the team in Nepal were particularly conscious that front-line public health workers were unlikely to share their views of any inadequacies in NCD services openly when senior managers were present. These barriers to open communication were addressed through separate group discussions based on seniority to ensure important views and suggestions were not overlooked in the process. Similarly, as mentioned above in Nigeria, understanding the dynamics of the relationship between informal providers and the formal sector was essential for effective as well as transparent communication. Gender dynamics and social hierarchies also determined communication, for example in Bangladesh, mid-career female researchers reported challenges in arranging and conducting meetings with senior, male government officials, and these interactions worked best when conducted by the most senior researchers, male or female. Recognition of these power dynamics was key to effective communication and rapport building.

The teams identified common challenges in working with government bureaucracies. Changing staff and particularly the rotation of senior decision-makers across leading government roles meant the teams were required to rebuild rapport and ensure those at the top of the hierarchy were still supportive of the initiative. For example, during the intervention design period, the team in Pokhara saw four different directors of the city government, and in Bangladesh, a change in government over the intervention period led to considerable change of senior government personnel. The teams soon learnt to keep communication channels open, particularly with lower cadres of government staff. In Ghana, the development of training and health promotion materials for the intervention was expected to be a rapid co-production exercise (see Table 2). However, it took nearly 10 months from the initial interactions with the technical working group/health promotion Teams to the final approval of materials. Allowing sufficient time and resources within the budget to maintain government engagement and support despite frequent changes and long processes has to be part of initial research proposal development.

#### Resultant initiative, value creation and potential outcomes

Within the intervention design process, the tension between developing a feasible health system intervention, the wide-ranging and extensive needs of communities and the desire of health professionals and mangers to deliver broad improvements across the system was more difficult to address. However, ultimately, the insights from these tensions led to the development of interventions that were more cognisant of the wider system. For example, in Nepal, while government actors were understandably keen to strengthen public primary care, and to take a system perspective during the design process, all participants were able to identify the potentially negative feedback-loop of referring pharmacy clients to an unprepared public primary care service for their diabetes and hypertension care. This led to the inclusion of capacity-strengthening activities for public health providers as well as pharmacy staff as a key component of the health systems intervention.

In Nigeria, the engagement of community leaders and the value placed on the lived experience of slum-dwellers throughout the design process underlined the need to include all types of informal providers—patent medicine vendors, bonesetters, TBAs and faith healers who were used extensively by all ages, genders, ethnicities and for multiple health conditions. Including all types of informal providers in the subsequent system-linkage intervention was key to maintaining trust in the community and building a foundation of quality and equity within the informal settlements. The needs assessment in Bangladesh highlighted the exclusion of the third gender from primary care, leaving them dependent on one or two pharmacies that were willing to serve them. The deep and wide changes required to overcome the stigma facing the third gender were hard to address in design process; however, the shared awareness of this issue did enable the team to include a third category under the gender field in the Simple App. As this revised version of the application is used across Bangladesh, this means that for the first time, data on the use (or lack of it) of primary care by the third gender will be recorded. This has the potential to bring this issue to the attention of policy makers for future health system change. In Ghana, the participatory nature of the initial needs assessment identified the multiple health needs of urban populations. This reinforced the need for a multipronged systems intervention to strengthen and expand existing services—such as the need to capacitate CHOs/Ns to deliver care for NCDs and to adapt outreach to communities to urban environments.

## Discussion

We found examples of co-production, co-design and co-creation within each development process. Categorizing a process as only one of these was inappropriate, as different characteristics of each approach were observed with certain groups of stakeholders at specific times during the process. Deep and embedded relationships with government stakeholders, particularly as so-called insider-researchers, has been identified as a key factor in supporting sustainable delivery of intervention [57, 58]. The approach of the HERDi team in embedding researchers within the Pokhara city authorities (PMC) could fit within this category and was clearly identified by the team as a facilitating factor in achieving government ownership, even when the intervention took an initially challenging direction of focusing on linking private pharmacies to the public system. Similarly, in Ghana, the district directors of health from the two intervention districts were also embedded within the research team contributing to planning and shaping the needs assessment and co-design phases. They played a significant role in identifying relevant stakeholders and assisting the team in understanding the context and appropriately engaging with different stakeholder groups.

Shaping interventions and recognizing their interaction within the complexity of health systems has also been identified as facilitating scale-up [59]. Facilitating and allowing the voices of multiple stakeholders is clearly a vital first step in understanding the complexity of the system [21, 25]. In Ghana, researchers used a systems approach to identify the research problem together with multiple stakeholders (city actors, health professionals and communities). This approach included drawing rich pictures and later analysing them to create interlinkage diagraphs and causal loop diagrams; it enabled the team to focus the intervention on specific, key elements of the health system. The careful consideration of potential feedback loops was also key to the development of the intervention in Nepal, where the evidence of limited preparedness to manage NCDs within the public system could have otherwise undermined referrals from pharmacies.

Co-creation was more likely to be achieved where researchers had long-standing relationships with stakeholders; this was common with national and city government actors. Where these long-standing relationships exist, it is hard to identify when the co-creation process begins. For example, in Bangladesh, the focus on NCDs emanates from long-standing concerns of ministry and previous work led by the research team in Bangladesh, the ARK Foundation [60]. Prioritization exercises conducted separately among community members, front-line and more senior health professional were needed to counter hierarchies. These findings chime with sociological perspectives which focus on the social relations and power between stakeholder groups [61]. Considering and reflecting on community and systems structures and the agency of stakeholder groups when preparing and implementing co-design processes was a key element for all the teams.

Establishing co-creation with communities was more challenging, with co-production where communities shared experiences and then gave feedback on existing prototypes of materials as the dominant form of engagement. Only when researchers made a conscious plan, often through the use of participatory methods, did community members become more engaged in shaping fundamental aspects of the health system interventions. The complexities and diversity of urban communities and the invisibility of several of the most vulnerable residents [62] presented the teams with dilemmas on who to include and how. Co-creation with communities was best achieved when the needs assessments included participatory methods, as was the case in Ghana and Nepal. However, where communities faced severe discrimination and exclusion, as with the hijra community in Bangladesh, the team used qualitative methods to explore their perspectives and feed these into the co-design process. Working with community leaders was vital, particularly in the informal settlements in Nigeria, where, as in other similar contexts [63], leaders play a crucial role in access and security. A limitation of our approach to reflection on the co-design process is that our reflective workshops only included the research teams involved in the process. This was due to the limitations of grant timelines and resources; however, the lack of reflections from community representatives or health system actors may have limited our insights and lessons learned on the process.

Long-standing unease at working with the profit-motivated private sector was an underlying value influencing public sector engagement, particularly at city level; however, the value placed on evidence and data as well as conducive policy environments helped balance these concerns. The need to include relevant evidence within the co-design process has been identified as key to successful public health intervention development [64, 65]. We found that where this evidence is clearly locally generated and relevant, it was particularly influential. That can be seen in the experience in Nepal where both quantitative as well as qualitative evidence was vital to challenge the public sector’s reluctance to work with the private sector in Nepal. This learning highlights the importance of researchers not only in sharing national or even global evidence but also in working with local stakeholders to design studies to generate local, contextually appropriate evidence and their role during the process of negotiating and balancing the evidence with views of stakeholders. The importance of contextual information and local needs assessments has been identified as a significant facilitator in the co-design of feasible and acceptable interventions [15].

Ultimately, the success of an intervention design process must be judged by the extent the intervention can be implemented successfully [66]. Within the context of improving public health and reducing inequities, successful implementation must be viewed as implementation that can reach those it targets, in this case, low-income urban residents, and can be adopted and implemented sustainably within routine practices and, ultimately, is effective in improving health outcomes. These elements are well considered within the RE-AIM framework [67] which will guide the planned evaluations of the implementation of the interventions presented here. Connecting the characteristics of the design process to the ultimate ability of the interventions to meet each aspect of the successful implementation is less studied. While reviews of the scale-up public health interventions offer valuable insights [57, 58], this is an area for exploration in further research.

## Conclusions

Co-creation in rapidly urbanizing contexts with multiple providers, diverse communities and fragmented governance arrangements is complex and requires considerable time, flexibility and on-going reflection. While a highly participatory co-creation process is desirable, in practice, the relationship between stakeholders in the design process oscillates between highly and less-engaged; multiple strategies appropriate to each stakeholder group are required at different timepoints. Careful consideration of the hierarchies among professionals and relationships between providers need to be taken into consideration throughout the process. This is particularly important when co-creation processes bring together public, private and informal providers where the public sector may have concerns regarding the private sector’s profit-motivation, while the private sector is, in turn, concerned about regulation and impact on their business. Allowing time, flexibility and carefully planning separate and joint interactions between the sectors can help to build trust. The diversity of urban communities and the long-working hours of low-income urban residents pose particular challenges in co-creation. An initial needs assessment to understand this diversity and to seek out experiences from vulnerable communities proved vital when direct engagement in the co-creation process could not be achieved. Targeted use of locally generated qualitative and quantitative information to highlight gaps and strengths in current services when planning intervention design processes was a key component of the co-creation process in these urban contexts where there are considerable gaps in existing data.

## Supplementary Information

Additional file1

## Data Availability

No datasets were generated or analysed during the current study.
