# Supplementary material for: Co-creation, co-design or co-production? Reflections on the development of urban health systems implementation strategies to improve access and quality of primary healthcare services in Bangladesh, Ghana, Nepal and Nigeria
Source: Health Res Policy Syst. 2026 Apr 27;24:45. doi: 10.1186/s12961-026-01467-4 (PMC13196040; doi:10.1186/s12961-026-01467-4)
Supplement: Supplementary file 1 — Additional file1 [file 12961_2026_1467_MOESM1_ESM.docx]

|  | **BANGLADESH** | | |
| --- | --- | --- | --- |
|  | **Activity** | **Date** | **Purpose** |
| **Preparatory phase** | Informal discussions with existing community groups, local city government and MoHFW, NCD department | 2020 | To shape the focus of the health systems intervention |
|  | Analysis of secondary data- (Bangladesh Health Facility Survey) and literature review of NCD primary care interventions. | 2021 to2022 | To understand current NCD service provision and identify potential health systems interventions for prevention and care of NCDs |
|  | Community advisory panels of community leaders and people with diabetes and hypertension meeting regularly | April 2022 onwards | To gain community insights on the focus and design of the intervention |
|  | Steering committee of national and city government, health providers, WHO, NGOs, urban health experts/academics meeting formally with informal meetings as needed with specific members | July 2022 onwards | To build ownership and ensure the evolving intervention was embedded within health systems structures, making use of existing initiatives (e.g. simple app.) to enable sustainable integration of the intervention within the system. |
|  | Stakeholder mapping across health providers (NGO and government), donor partners, national and local government and community groups. | April – June 2022 | To identify individuals and organisations involved in urban primary care and NCD prevention and care to inform qualitative sampling and engagement in codesign workshops |
|  | Qualitative interviews and primary health care facility assessment | May – August 2022 | To understand current NCD service provision and experience of disadvantaged communities and people living with NCDs |
| **Co-design phase** | Workshop 1: Sharing needs assessment, secondary analysis and literature review findings and problem and solution prioritization with national, city governments, urban health experts, NGOs and community advisory panels (one day, 42 participants) | September 2022 | To ensure problem and solution prioritisation were grounded in existing evidence |
|  | Workshop 2: Specifying the intervention with national, city governments, urban health experts, NGOs and community advisory panels (one day, 38 participants) | October 2022 | To agree the specifics of the intervention including content of diabetes and hypertension services, roles of primary care providers in both NGO and government dispensaries, fields for simple app and linkage with DHIS2 and training needs. |
|  | Meetings/Workshop 3: Feedback on material design from community advisory panel (3 male, 5 female) and health providers (5) | February 2023 | To fine-tune the intervention materials based on practical insights from health providers and people with hypertension and diabetes. |
|  | Implementation begins with training of health providers | October 2023 |  |
|  | **GHANA** | | |
|  | **Activity** | **Date** | **Purpose** |
| **Preparatory phase** | Informal meetings with district health teams and Ghana Health Service | 2020 | To understand district challenges with CHPS and agree the process for codesign |
|  | 4 Rich picture workshops: 2 in each district, first with managers and then with community health workers and community members | 2021 | To understand system-wide challenges to the CHPS programme |
|  | 2 transect walks with community health workers and community members | 2021 | To understand health seeking behaviour and challenges with CHPS in each community |
|  | Households survey in both districts; qualitative interviews and focus groups with community members, community health workers and managers | 2021-2023 | To understand and quantify health needs and health seeking behaviour and experiences of CHPS |
|  | Establishment and then ongoing meetings with technical advisory groups in each community and with district health teams | 2022 | To build ownership and ensure the evolving intervention was embedded within health systems structures for sustainable integration of the intervention within the system. |
| **Codesign phase** | **Co-creation workshop (Ashaiman)**  16 Technical Advisory Group members  (6 females, 10 males) (One day) | Sept  2023 | To disseminate needs assessment findings and seek stakeholders' understanding of proposed interventions and their reasons for accepting or rejecting any proposed interventions. |
|  | **Co-creation workshop (Madina)**  16 Technical Advisory Group members  (6 females, 10 males) (One day) | Oct  2023 | To disseminate needs assessment findings and seek stakeholders' understanding of proposed interventions and their reasons for accepting or rejecting any proposed interventions. |
|  | **Consumer Dipstick of health promotion materials (Madina)**  Residents of the intervention sites (Two FGDs were conducted with a total of 18 women and 10 men in this community) (One day) | July 2024 | The consumer dipstick aimed to assess the clarity, suitability, and cultural relevance of the materials. It evaluated audience perceptions, checked for potentially offensive content, and examined whether the language and visuals were appropriate and easily understood, while also gathering suggestions for improvement. |
|  | **Consumer Dipstick of health promotion materials (Ashaiman)**  Residents of the intervention sites (Two FGDs were conducted with a total of 10 women and 13 men in this community) (One day) | July 2024 | The consumer dipstick aimed to assess the clarity, suitability, and cultural relevance of the materials. It evaluated audience perceptions, checked for potentially offensive content, and examined whether the language and visuals were appropriate and easily understood, while also gathering suggestions for improvement. |
|  | Finalisation of all materials and training of CHOs and CHNs | September 2024 | To finalise all health education materials and the training package for use within the intervention and for wider dissemination. |
|  | **NEPAL** | | |
|  | **Activity** | **Date** | **Purpose** |
| **Preparatory phase** | Analysis of urban population data (n = 3460) from Nepal’s STEP survey (2019) | 2020-2021 | To identify the influence of social determinants and poverty with NCD risk factors and prevalence in urban areas to inform the focus of the intervention to be codesigned with pharmacists, communities, public healthcare services and Pokhara Metropolitan City |
|  | Rapid review of federal and provincial urban health, pharmacy and NCD policies | 2021 | To identify any policy and legal frameworks relating to the role of pharmacies and partnerships between public and private health providers/pharmacies. |
|  | Rapid review of NCD and primary care studies conducted in Nepal | 2021 | To identify lessons learnt on strengthening NCD prevention and care within the public and private primary care sector. |
|  | Reconnaissance informal meetings and then participatory approaches with community gatekeepers including two social mapping exercises and transect walks. | 2022-2023 | To build rapport with communities in deprived neighbourhoods and understand their health seeking behaviour within local pharmacies, primary, secondary and other providers. |
|  | Qualitative methods of 7 interviews with city officials and health providers, 6 focus groups with community members and 10 interviews with NCD patients | 2023 | To understand perspectives of the role of pharmacies and opportunities and motivation for pharmacists’ role in NCD prevention and care within the public health system. |
|  | Cross-sectional assessment of 398 primary care and pharmacy NCD services. (31) | 2023 | To assess current practices in relation to NCDs and any existing linkages between pharmacies and the formal health care system. |
| **Co-design phase** | Workshop with community female members  (14 female) (One day) | 13 Jan 2023 | To understand health problems, health seeking behaviour in low-income urban neighbourhoods and possible solutions (using problem tree analysis of causes) |
|  | Workshop with community male members  (13 male) (One day) | 13 Jan 2023 | To understand health problems, health seeking behavior in low-income urban neighbourhoods and possible solutions (using problem tree analysis of causes) |
|  | Workshop with Community health volunteers  (9 female) (One day) | 14 Jan 2023 | To understand health problems, health seeking behavior in low-income urban neighbourhoods and possible solutions |
|  | Workshop with Community health volunteers  (8 female) (One day) | 14 Feb 2023 | To understand health problems, health seeking behavior in low-income urban neighbourhoods and possible solutions |
|  | Working with ward 1: Health Facility Operation and Management Committee (HFOMC) members and ward chairs (3 female, 6 male) | 23 Feb 2023 | To share findings of the need assessment and discussion solutions regarding NCD service improvement across public and private providers |
|  | Workshop with public health care providers  (2 male, 7 female) (one day) | 27 Feb 2023 | To identify potential challenges of NCD service provision and linkages with pharmacies from the perspective of the health care providers and generate the solutions |
|  | Ward 2: Health Facility Operation and Management Committee (HFOMC) members and ward chairs (2 female, 9 male) (One day) | 3 March 2023 | To share findings of the need assessment and discussion solutions regarding NCD service improvement across public and private providers |
|  | Workshop with female group with NCDs (8 female) (One day) | 10 Sept 2023 | To understand health problems, health seeking behavior in low-income urban neighbourhoods and possible solutions (using problem tree analysis of causes) |
|  | Consultative meeting with Health Division (3 male, 1 female) | 21 Sept 2023 | To understand challenges of health system to collaborate with private sector and possible ways to engage them |
|  | Workshop with pharmacists (5 male) (One day) | 21 Sept 2023 | To understand service practices of pharmacies, discuss and identify any potential linkage with wider health system |
|  | Workshop with ward elected representatives (4 male, 1 female)  (One day) | 22 Sept 2023 | To share findings of need assessment and understand their opinion on potential linkage between pharmacies and wider health system |
|  | Consultative meeting with Sisuwa primary hospital (3 male, 2 female) | 22 Sept 2023 | To discuss ways to create referral linkage between pharmacies and health and primary hospital |
|  | **NIGERIA** | | |
|  | **Activity** | **Date** | **Purpose** |
| **Preparatory phase** | Scoping review of interventions connecting informal providers with formal primary care (10) | 2020 - 2022 | To identify lessons learnt from previous programmes in sub-Saharan Africa to inform the co-design of the health systems linkage strategies. |
|  | Rapid review of Enugu State policies | 2020 - 2021 | To ensure the strategies for linking the informal providers to the formal public health-care system were aligned with current policy, particularly in relation to any limitations to the roles of informal providers. |
|  | Reconnaissance in the four informal settlements including introductory meetings followed by qualitative interviews with 32 informal providers and 16 community leaders (11). | 2021 – 2022 | To build rapport with community members and understand their perspectives on the role of informal and formal providers in informal settlements. |
|  | Cross-sectional assessment of 254 formal and informal health facilities in 8 slums across 2 states and 1025 households to assess use of formal and informal providers (32) | 2022 – Jan 2023 | To assess current practices, including any existing referral or other linkages between formal and informal providers. |
| **Co-design phase** | **Co-creation workshop 1** (with State & LGA policymakers, formal and informal providers, community representatives, experts, 35 participants) | 15-16 Feb 2023 | To invite stakeholders to reflect on findings from the baseline assessment, and based on this to prioritize potentially feasible interventions and develop operational plans for implementing the interventions |
|  | **Co-creation workshop 2**  (same categories of participants as workshop 1, mostly the same participants but some organisations sent other team members due to availablity) | 25 May 2023 | To review and validate the logic framework for each intervention and develop outlines of protocols and tools for implementation. |
|  | **Co-creation workshop 3** (researchers only) | 21-23 Feb 2024 | To further develop plans, protocols, including measurements to implement interventions |
